# Publisher Correction: Standardized preservation, extraction and quantification techniques for detection of fecal SARS-CoV-2 RNA

**DOI:** 10.1038/s41467-021-27392-4

**Published:** 2021-12-01

**Authors:** Aravind Natarajan, Alvin Han, Soumaya Zlitni, Erin F. Brooks, Summer E. Vance, Marlene Wolfe, Upinder Singh, Prasanna Jagannathan, Benjamin A. Pinsky, Alexandria Boehm, Ami S. Bhatt

**Affiliations:** 1 Department of Genetics, Stanford University, Stanford, CA USA; 2 Department of Medicine (Hematology, Blood and Marrow Transplantation), Stanford University, Stanford, CA USA; 3 Department of Microbiology and Immunology, Stanford University, Stanford, CA USA; 4 Department of Civil and Environmental Engineering, Stanford University, Stanford, CA USA; 5 Department of Medicine (Infectious Diseases and Geographic Medicine), Stanford University, Stanford, CA USA; 6 Department of Pathology, Stanford University, Stanford, CA USA

**Keywords:** Reverse transcription polymerase chain reaction, Pathogens, Translational research, Reverse transcription polymerase chain reaction, Pathogens, Translational research

Correction to: *Nature Communications* 10.1038/s41467-021-25576-6, published online 1 October 2021.

The wrong Supplementary file was originally published with this article; it has now been replaced with the correct. The original article has been corrected.

## Supplementary information

Updated Supplementary Information

