# Supplementary material for: Standardized preservation, extraction and quantification techniques for detection of fecal SARS-CoV-2 RNA
Source: Nat Commun. 2021 Oct 1;12:5753. doi: 10.1038/s41467-021-25576-6 (PMC8486790; doi:10.1038/s41467-021-25576-6)
Supplement: Supplementary file 1 — Supplementary Information [file 41467_2021_25576_MOESM1_ESM.pdf]

Supplementary Information file

Standardized preservation, extraction and quantification techniques for detection of fecal SARS-CoV-2 RNA

Aravind Natarajan[1,2#], Alvin Han[3#], Soumaya Zlitni[1,2], Erin F. Brooks[2], Summer E. Vance[2], Marlene Wolfe[4], Upinder Singh[5], Prasanna Jagannathan[3,6], Benjamin A. Pinsky[5,7], Alexandria Boehm[4], Ami S. Bhatt[1,2]*

[1] Department of Genetics, Stanford University, Stanford, CA, USA.
[2] Department of Medicine (Hematology, Blood and Marrow Transplantation), Stanford University, Stanford, CA, USA.
[3] Department of Microbiology and Immunology, Stanford University, Stanford, CA, USA.
[4] Department of Civil and Environmental Engineering, Stanford University, Stanford, CA, USA.
[5] Department of Medicine (Infectious Diseases and Geographic Medicine), Stanford University, Stanford, CA, USA.
[6] Department of Medicine (Infectious Diseases), Stanford University, Stanford, CA, USA.
[7] Department of Pathology, Stanford University, Stanford, CA, USA

[#] These authors contributed equally
* Address correspondence to: Ami S. Bhatt, 269 Campus Dr, CCSR 1155b, Stanford University, Palo Alto, CA 94305. Tel: (650) 498-4438; Email: asbhatt@stanford.edu.

Supplementary Figures

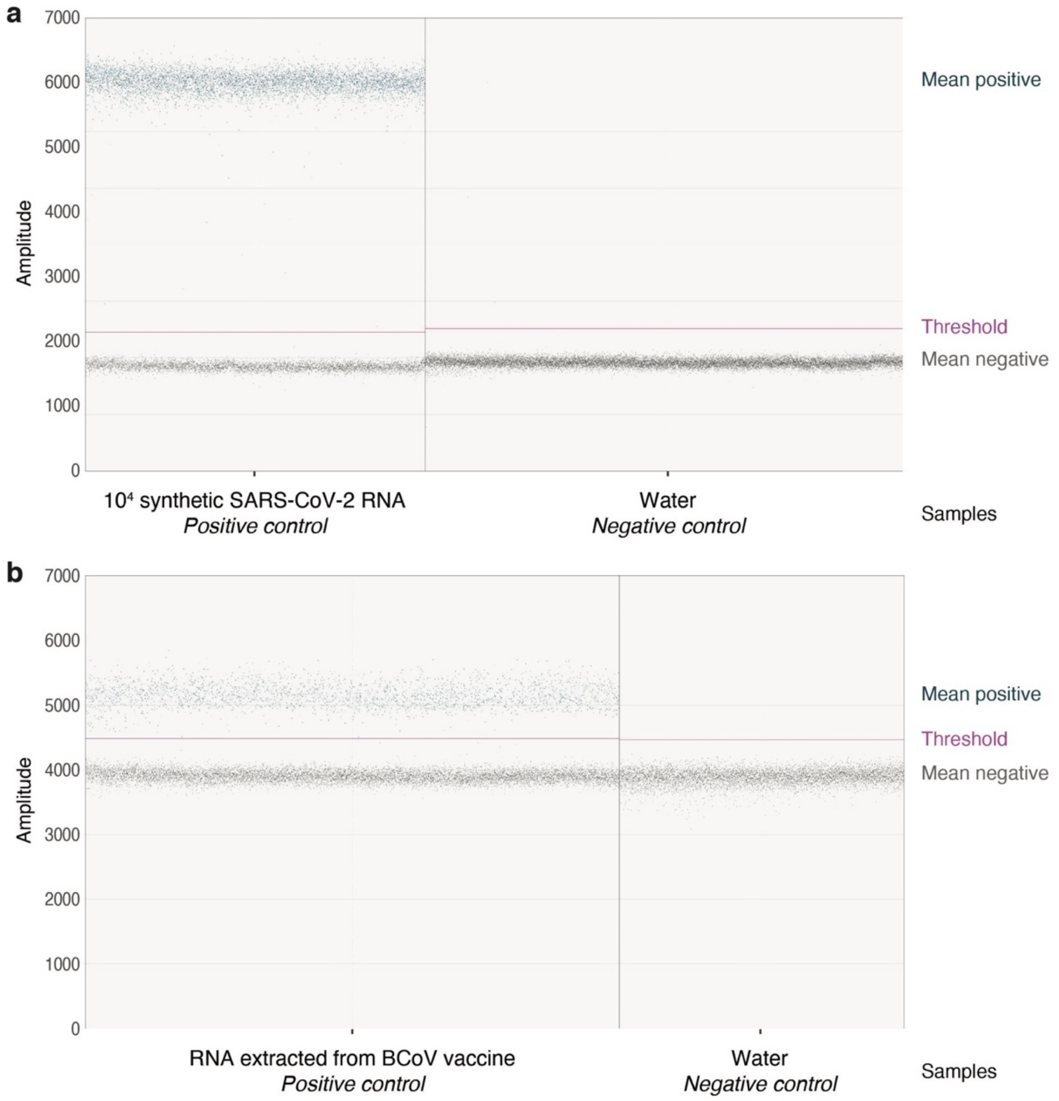

Supplementary Fig. 1 Examples of thresholding of positive and negative ddPCR reactions. Dot plots present amplitude of fluorescence intensity on the y-axis and sample source on the x-axis. Each dot represents a single droplet partition in a given assay. Mean positive amplitude, threshold amplitude and mean negative amplitudes are marked to the right of the plots. Threshold for each sample is also marked by a purple line across the plots. a. Example reactions from assays targeting the SARS-CoV-2 N1 gene. Plot to the left captures data from an assay of the ATCC synthetic SARS-CoV-2 RNA that serves as a positive control, and plot to the right captures water, the negative no template control. b. Example reactions from assays targeting the BCoV M gene. Plot to the left captures data from an assay of RNA extracted using the Zymo Quick-RNA Viral Kit from the BCoV vaccine that serves as a positive control, and plot to the right captures water, the negative no template control.

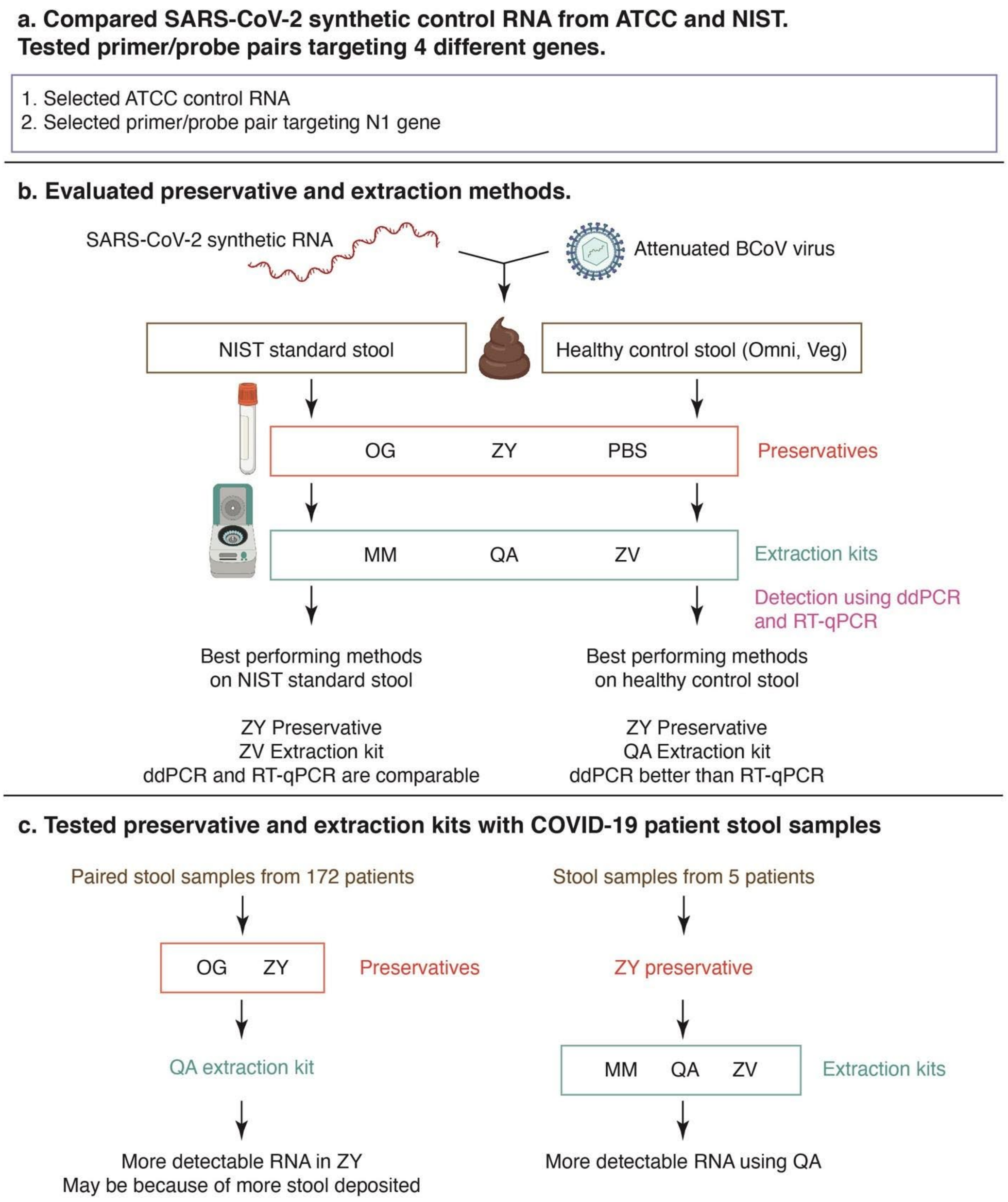

Supplementary Fig. 2 Schematic illustration of key experiments and results. a. Comparisons of SARS-CoV-2 synthetic control RNA standards from ATCC and NIST revealed that ATCC performed reliably. Further, the primer/probe set targeting the N1 gene in SARS-CoV-2 RNA performed reliably at detecting viral RNA. b. Standardized stool samples from NIST, and non-standardized stool samples from two healthy donors, one on an omnivorous diet and one on a vegetarian diet, were employed to evaluate various preservatives and extraction methods. Stool samples were spiked with synthetic SARS-CoV-2 RNA or attenuated BCoV vaccine at defined concentrations. Samples were preserved in the OG, ZY or PBS buffers, and viral RNA was extracted using the MM, QA, or ZY kits. Viral RNA was quantified using ddPCR and RT-qPCR targeting the N1 gene in SARS-CoV-2 or the M gene in BCoV. In NIST samples, ZY and ZV performed best while in healthy stool samples, ZY and QA performed best. Although both ddPCR and RT-qPCR performed comparably at detection RNA extracted from NIST samples, ddPCR performed better in RNA extracted from healthy stool samples. c. Performance of the preservatives and extraction kits were assessed using clinical samples. In the first experiment (left) viral RNA extracted using QA from 172 paired patient stool samples preserved in the OG and ZY buffers was analyzed. ZY preserved samples yielded more detectable RNA, perhaps because more stool was deposited in this kit. Next, in the second experiment (right) viral RNA was extracted from 5 patient stool samples preserved in ZY using MM, QA and ZV. Here, QA yielded more detectable RNA.

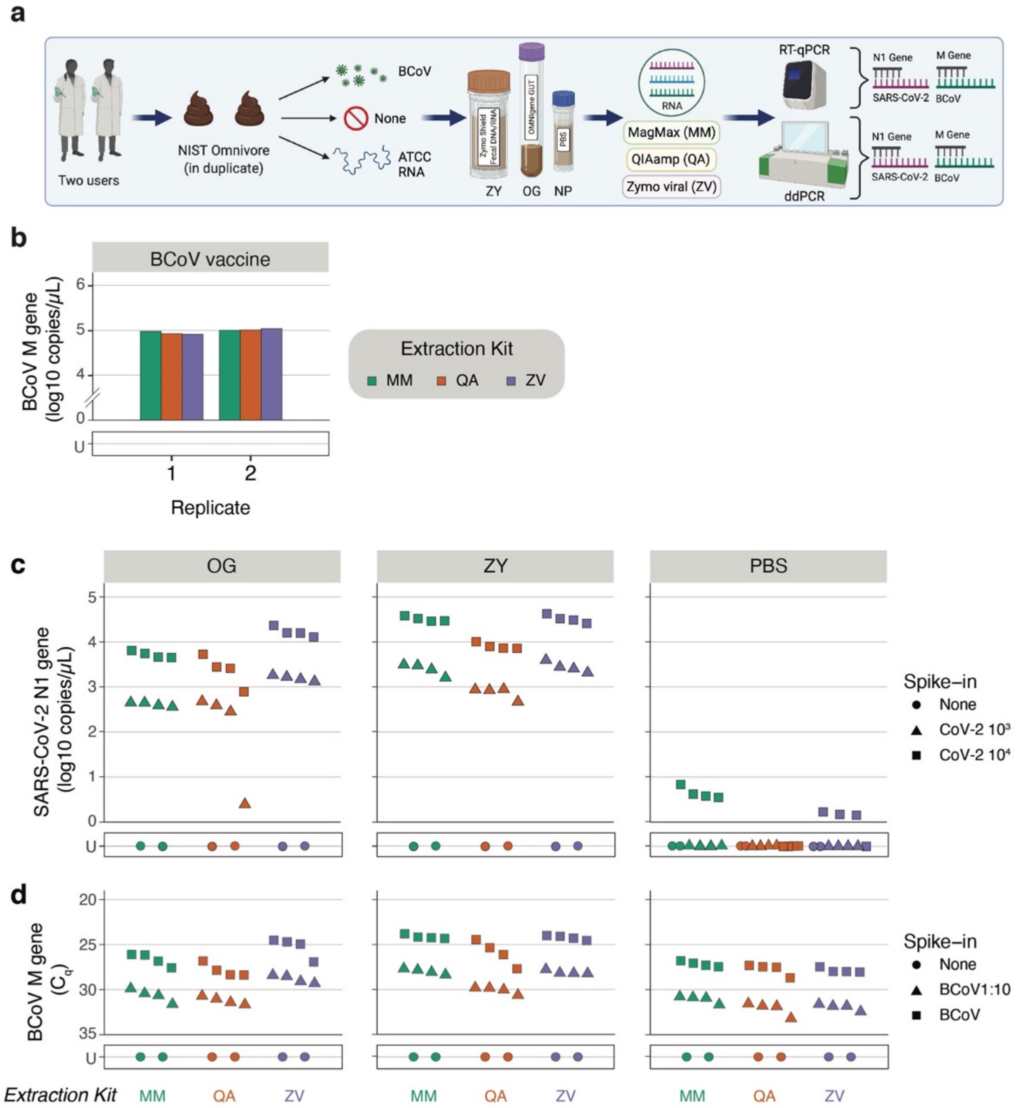

Supplementary Fig. 3 Efficacy of preservation and RNA extraction of SARS-CoV-2 and BCoV RNA from standardized NIST stool. a. Two independent users performed this experiment in duplicate. Stool samples collected from omnivorous donors and processed into a standardized matrix by NIST were spiked with ATCC CoV-2 RNA, a BCoV vaccine or equal volume of PBS (no RNA). Spiked stool was preserved in the OMNIgene-GUT kit (OG), Zymo DNA/RNA shield buffer (ZY) and PBS. RNA was extracted from these samples using the MagMAX Viral/Pathogen Kit (MM), QIAamp Viral RNA Mini Kit (QA) or Zymo Quick-RNA Viral Kit (ZY). RNA was assayed using ddPCR and RT-qPCR targeting the SARS-CoV-2 N1 gene or BCoV M gene. b. As an extraction control, RNA was isolated from the BCoV vaccine directly without the stool matrix using MM (green), QA (orange) and ZY (purple) kits. Each user included a set of these extractions (indicated in the x-axis). Absolute concentration of BCoV RNA assayed by ddPCR targeting the M gene is plotted on the y-axis. c. RNA extracted using the MM (green), QA (orange), and ZY (purple) kits are listed on the x-axis, and concentration of SARS-CoV-2 RNA assayed by RT-qPCR targeting the N1 gene is plotted on the y-axis. NIST stool matrix was spiked with 10^3 (triangle) or 10^4 (square) copies of ATCC synthetic SARS-CoV-2 RNA and processed in quadruplicate. d. RNA extracted using the MM (green), QA (orange) and ZY (purple) kits are listed on the x-axis, and C_q value of RT-qPCR assays targeting the BCoV M gene is plotted on the y-axis. NIST stool matrix was spiked with 1:10 diluted (triangle) or undiluted (square) BCoV vaccine. Control samples with no spiked in RNA (none; circle) were included in duplicate to estimate LoB. U stands for undetermined and marks samples with no detectable RNA above LoB. RT-qPCR assays were run in technical duplicates and the mean values are represented here. Source data are provided as a Source Data file.

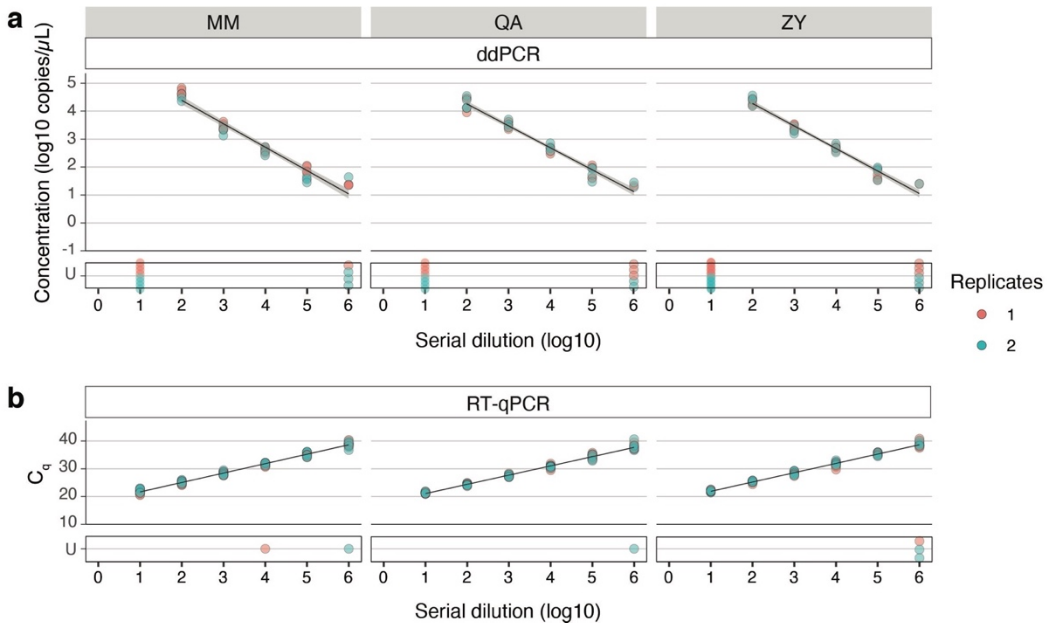

Supplementary Fig. 4 Robustness of primer/probe set at quantifying BCoV. ddPCR and RT-qPCR assays targeting M gene from BCoV across a seven-point ten-fold dilution series of RNA extracted from BCoV vaccine. RNA was extracted using either the MagMAX Viral/Pathogen Kit (MM), QIAamp Viral RNA Mini Kit (QA) or Zymo Quick-RNA Viral Kit (ZY) as indicated on the tab to the top. a. Dilutions of RNA are plotted on the x-axis and absolute copy number derived from ddPCR is plotted on the y-axis. All assays were performed in duplicate. b. Dilutions of RNA are plotted on the x-axis and C_q derived from RT-qPCR is plotted on the y-axis. All assays were performed in quadruplicate. Replicates in red and blue refer to two independent experiments performed by two users using separate extractions of RNA. Linear regression is plotted in black and 95% confidence interval is shaded in gray. Samples that did not amplify are delineated as U for undetermined and not included in the linear regression analysis. Associated statistics are summarized in Supplementary Table 1. Source data are provided as a Source Data file.

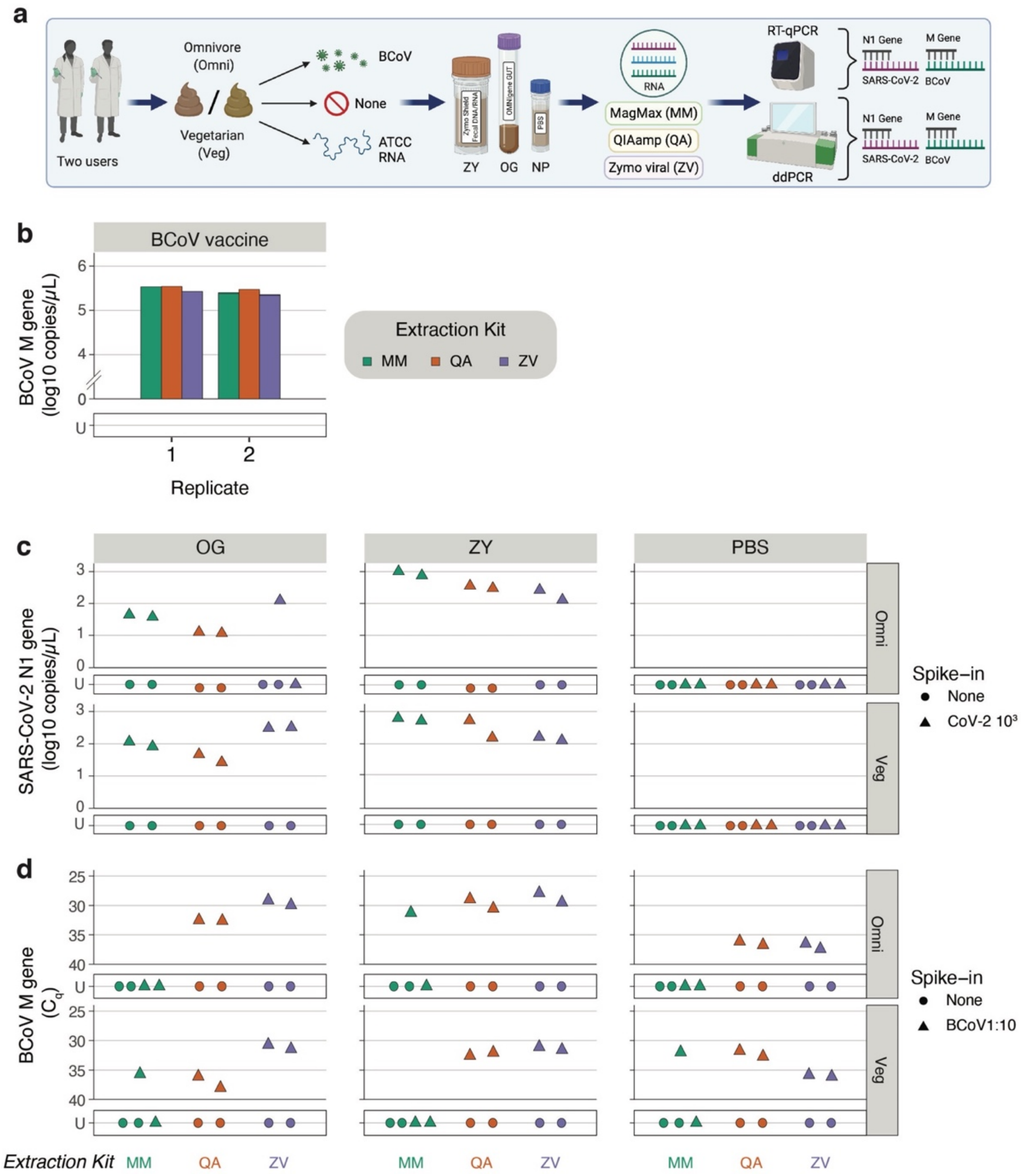

Supplementary Fig. 5 Performance of preservation and extraction of SARS-CoV-2 and BCoV RNA from non-standardized stool samples from healthy donors. a. Two independent users performed this experiment. Stool samples were collected from healthy omnivorous (Omni) and vegetarian (Veg) donors and spiked with ATCC CoV-2 RNA or BCoV vaccine or equal volume of PBS (no RNA). Spiked stool was preserved in the OMNIgene-GUT kit (OG), Zymo DNA/RNA shield buffer (ZY) and PBS. RNA was extracted from these samples using the MagMAX Viral/Pathogen Kit (MM), QIAamp Viral RNA Mini Kit (QA) or Zymo Quick-RNA Viral Kit (ZY). RNA was assayed using ddPCR and RT-qPCR targeting the SARS-CoV-2 N1 gene or BCoV M gene. b. As an extraction control, RNA was isolated from the BCoV vaccine directly without the stool matrix using MM (green), QA (orange) and ZY (purple) kits. Each user included a set of these extractions (indicated in the x-axis). Absolute concentration of BCoV RNA assayed by ddPCR targeting the M gene is plotted on the y-axis. c. Concentration of SARS-CoV-2 RNA assayed by RT-qPCR targeting the N1 gene is plotted on the y-axis. Healthy stool samples were spiked with 10^3 (triangle) copies of ATCC synthetic SARS-CoV-2 RNA. d. C_q values from RT-qPCR assays of BCoV RNA targeting the M gene are plotted on the y-axis. Healthy stool samples were spiked with 1:10 diluted (triangle) BCoV vaccine. Control samples with no spiked in RNA (none; circle) were included in duplicate to estimate LoB. U stands for undetermined and marks samples with no detectable RNA above LoB. Source data are provided as a Source Data file.

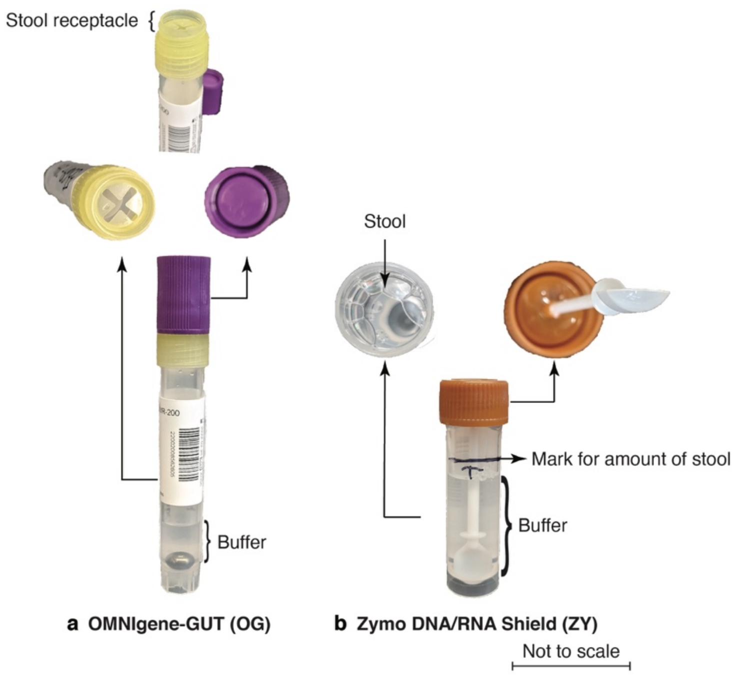

Supplementary Fig. 6 Photographs of the OG and ZY collection and preservation kits a. The OMNIgene-GUT (OG) collection kit includes a special receptacle of defined volume for the collection of stool samples. This is followed by a tube containing 2 ml of proprietary preservative buffer and a metal ball for homogenizing the sample. b. Zymo DNA/RNA Shield (ZY) kit is a standard collection tube with the proprietary DNA/RNA shield buffer and plenty of room in the tube above the buffer level for collection of stool.

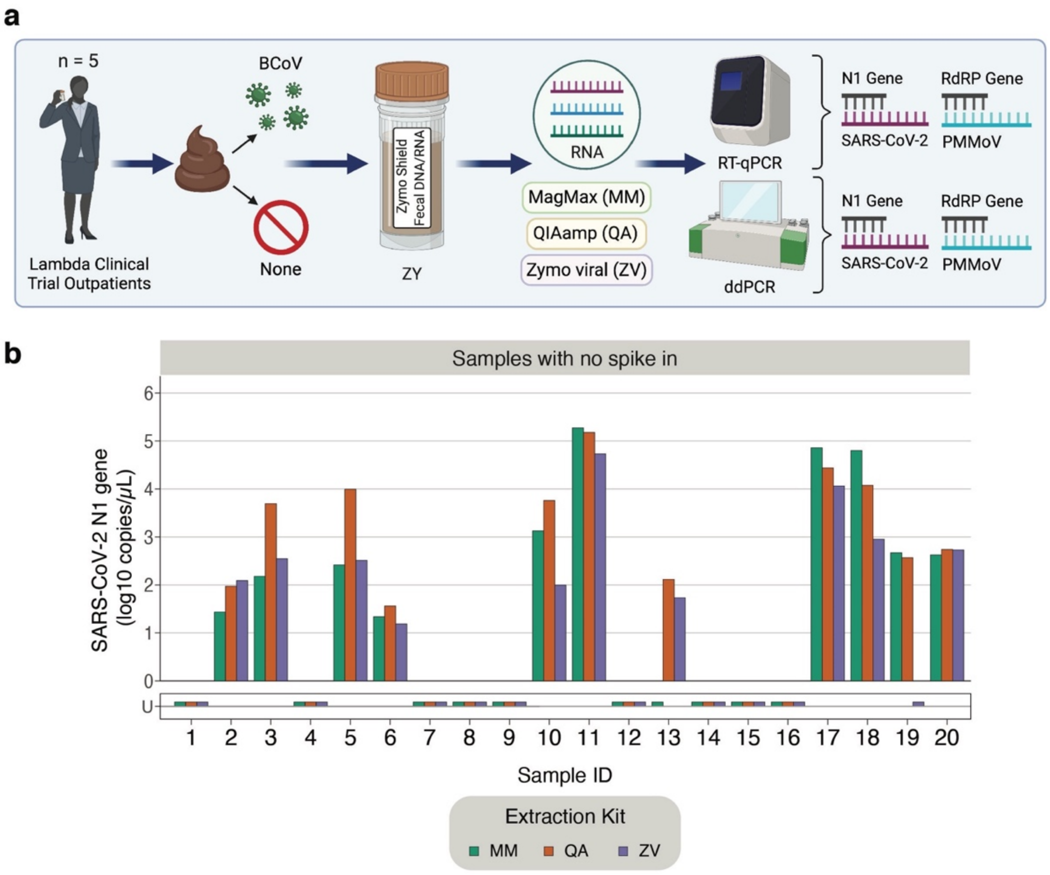

Supplementary Fig. 7 Testing efficiency of three extraction kits using clinical samples stored in the ZY preservative. a. Stool samples were collected in the Zymo DNA/RNA shield buffer (ZY) preservative from five COVID-19 outpatients enrolled in a clinical trial of Peginterferon Lambda-1a. All samples were spiked with 10 µl of undiluted BCoV vaccine. In parallel, the same set of samples were also processed without any spike-in. RNA from these samples were extracted using the MagMAX Viral/Pathogen Kit (MM; green), QIAamp Viral RNA Mini Kit (QA; orange) or Zymo Quick-RNA Viral Kit (ZY; purple). b. RNA from samples with no spike in were assayed for SARS-CoV-2 RNA using ddPCR targeting the N1 gene. Anonymized sample identities are listed on the x-axis and absolute concentration is listed on the y-axis. U stands for undetermined and marks samples with no detectable RNA above LoB. Source data are provided as a Source Data file.

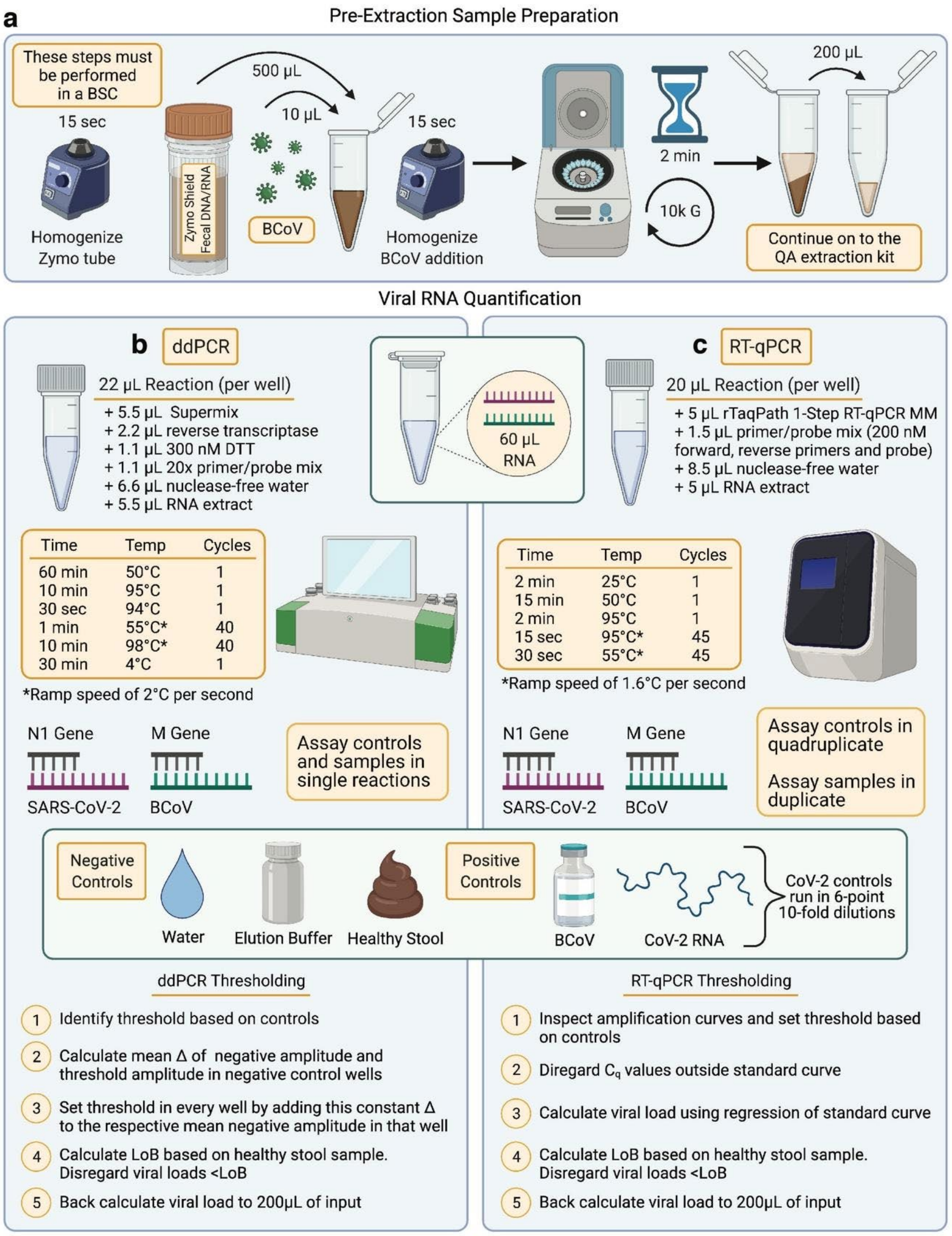

Supplementary Fig. 8 Recommended guidelines for the effective collection and preservation of stool samples for SARS-CoV-2 viral RNA extraction and detection in stool. Illustrated here are recommended guidelines for the detection of SARS-CoV-2 RNA from fecal samples. a. Pre-extraction sample preparation. ZY preservation kit is used for the collection of fecal samples from patients. The successive steps are carried out in a biosafety cabinet (BSC). ZY kit with stool is vortexed for 15 seconds. 500 µl of homogenized stool is transferred to an RNase free, sterile microcentrifuge tube, and spiked with 10 µl of BCoV vaccine. Sample is then vortexed for 15 seconds to ensure uniform mixing of the BCoV control. Spiked in stool aliquot is then centrifuged at 10,000 x g for 2 minutes and 200 µl of the clarified supernatant is transferred to a fresh microcentrifuge tube for RNA extraction using the QA kit as per manufacturer instructions. RNA is eluted in 60 µl of elution buffer. Viral RNA is quantified in this eluate using ddPCR and/or RT-qPCR as follows. b. Absolute concentration of RNA is assayed using the one-step RT-ddPCR advanced kit for probes as recommended[19]. Reaction constituents and thermocycling conditions summarized here can be applied to detect the SARS-CoV-2 N1 gene and BCoV M gene. Every assay plate should include three negative controls - water, elution buffer and viral RNA extracted from a healthy stool sample - to determine the Limit of Blank (LoB). Further, every assay plate should include positive controls - QA extracted RNA from BCoV and SARS-CoV-

2 synthetic RNA from ATCC. The mean positive and negative amplitudes from these controls are used to guide an appropriate threshold for analysis. The threshold is set between the mean positive and negative amplitudes, such that the negative control does not record presence of viral RNA, while the positive controls reflect the expected RNA concentration. Next, the mean difference between the mean negative amplitude and the threshold amplitude in the negative control reactions is calculated. This difference is added to the mean negative amplitude from every reaction in order to identify a normalized threshold for that specific reaction. c. Relative concentration of RNA is assayed using the TaqPath 1-Step RT-qPCR Master Mix, CG as recommended in the manufacturer protocol. Reaction constituents and thermocycling conditions summarized here can be applied to detect the SARS-CoV-2 N1 gene and BCoV M gene. Every 384-well assay plate should include control reactions in quadruplicate. This includes three negative controls - water, elution buffer and viral RNA extracted from a healthy stool sample - to determine the Limit of Blank (LoB). Further, every assay plate should include positive controls - QA extracted RNA from the BCoV vaccine, and a six-point ten-fold dilution series of SARS-CoV-2 synthetic RNA from ATCC starting at 10^4 copies/µl. Using the control reactions as a reference, inspect the amplification curves of the samples to ensure they are bonafide read-outs and establish a threshold. Disregard C_q values outside the standard curve as Undetermined since they cannot be used to accurately calculate the viral load. Using a linear regression of the synthetic RNA standards, calculate the relative concentration of viral RNA extracted from the stool sample. Across both ddPCR and RT-qPCR assays, disregard viral loads less than or equivalent to the LoB and back calculate the viral RNA load in the starting samples.

Supplementary Note 1

We have reported yields of viral RNA across all conditions in copies of target RNA per µL of stool supernatant. This has been the standard in studies analyzing the shedding of SARS-CoV-2 RNA in stool. However, the final target RNA concentration is dependent on the amount of stool collected. In fact, we see this influence of the amount of stool collected on target RNA yield in our comparison of paired clinical samples in the OG and ZY preservatives. Therefore, reporting yields of RNA normalized to input stool amount is the most universal metric, allowing comparisons both within and across studies. Hence, we translate the yields reported in this experiment in relation to the amount of stool collected using the following dimensional analysis:

*Total copies from input volume = Copies per µL in eluate * total eluant volume from extraction kit*

*Grams of stool from input volume = Percent stool in sample by weight * volume of input sample*

Copies per g of stool = Total copies from input volume / grams of stool from input volume

Description of variables and related assumptions

Copies per µL in eluate: Absolute concentration derived from ddPCR and normalized by total volume of the reaction (22 µL) and amount of eluate added to the reaction as template (5.5 µL). This assumes a 100% conversion of template RNA to cDNA in the one-step ddPCR reaction. We believe that this conversion is highly efficient, but likely not 100%.

Total eluant from extraction kit: Across samples and extraction kits, we eluted the RNA in 60 µL of elution buffer.

Volume of input sample: Across preservatives, we collected stool, homogenized the sample by vortexing, and aliquoted 200 µL of the clarified supernatant for extraction after centrifugation. Previous work on wastewater samples indicated that buffer solutions effectively liberate viral particles and RNA from solids[48]. Therefore, we assume that the virus like particle and viral RNA are fully released into the liquid supernatant of the sample.

Percent stool in sample by weight: Since the original biobanking effort was not designed to measure weight of stool collected, we had to find a rational method of calculating the percent stool in sample by weight, post-hoc. Ideally, we know that the ZY collection and preservation kit contains 9 mL of preservative. Further, by measuring the weight of the collection tube before and after collection, we could have been able to calculate the weight of stool deposited. However, in the absence of this information, we set about finding approximations in the following manner. We took two biopsy punches of frozen, homogenized stool and measured the wet weight. Next, we completely dried the stool sample and measured the corresponding dry weight. We then assumed that the dried biomass is the amount of stool deposited. The shortcoming of this assumption is that stool is not entirely dry and certainly adds liquid mass to the sample. However, we believe that this assumption will allow us to report concentration per amount of stool within an order of magnitude from the original concentration. Based on this assumption, we used the dry weight of stool in relation to the total wet weight to calculate the percentage of preserved sample constituted by stool.

Sample ID	Extraction	Copies per µL in eluate	Percent stool in sample by weight (%)	Stool weight estimate (g)	Copies per g stool
1	MM	0	34.3066	0.171533	0
1	QA	0	34.3066	0.171533	0
1	ZY	0	34.3066	0.171533	0
2	MM	27.2	33.7778	0.168889	24157.89
2	QA	94	33.7778	0.168889	83486.84
2	ZY	124	33.7778	0.168889	110131.6
3	MM	152	32.5	0.1625	140307.7
3	QA	4928	32.5	0.1625	4548923
3	ZY	352	32.5	0.1625	324923.1
4	MM	0	33.5714	0.167857	0
4	QA	0	33.5714	0.167857	0
4	ZY	0	33.5714	0.167857	0
5	MM	260	31.0127	0.155063	251510.2
5	QA	9840	31.0127	0.155063	9518694
5	ZY	324	31.0127	0.155063	313420.4

References:

42. qPCR efficiency calculator at https://www.thermofisher.com/us/en/home/brands/thermo-scientific/molecular-biology/molecular-biology-learning-center/molecular-biology-resource-library/thermo-scientific-web-tools/qpcr-efficiency-calculator.html
43. Corman, V. M. *et al.* Detection of 2019 novel coronavirus (2019-nCoV) by real-time RT-PCR. *Euro Surveill.* 25, (2020).
44. 2019-Novel Coronavirus (2019-nCoV) Real-time rRT-PCR Panel, (Centers for Disease Control, Atlanta, Georgia, 2020).
45. Loeb, S. One-Step RT-ddPCR for Detection of SARS-CoV-2, Bovine Coronavirus, and PMMoV RNA in RNA Derived from Wastewater or Primary Settled Solids. (2020).
46. Bustin, S. A. *et al.* The MIQE guidelines: minimum information for publication of quantitative real-time PCR experiments. *Clin. Chem.* 55, 611–622 (2009).
47. The dMIQE Group & Huggett, J. F. The Digital MIQE Guidelines Update: Minimum Information for Publication of Quantitative Digital PCR Experiments for 2020. *Clinical Chemistry* 66, 1012–1029 (2020).
48. Simpson, A., Topol A., White B., Wolfe M., Wigginton K., Boehm A.B.. 202X. Effect of storage conditions on SARS-CoV-2 RNA quantification in wastewater solids. Preprint at https://www.medrxiv.org/content/10.1101/2021.05.04.21256611v1. (2021)
