# Supplementary material for: Standardized preservation, extraction and quantification techniques for detection of fecal SARS-CoV-2 RNA
Source: Nat Commun. 2021 Oct 1;12:5753. doi: 10.1038/s41467-021-25576-6 (PMC8486790; doi:10.1038/s41467-021-25576-6)
Supplement: Supplementary file 9 — Description of Additional Supplementary Files [file 41467_2021_25576_MOESM9_ESM.pdf]

Title: Supplementary Data 1.
Description: Statistical measures from the regression of detection of synthetic SARS-CoV-2 viral RNA and BCoV RNA standards. Statistical tests are two-sided and made without adjustments for multiple comparisons.

Title: Supplementary Data 2.
Description: Paired two-sided t-tests to evaluate the significance of the differential performance of preservatives and extraction kits used with NIST stool samples. N = 4 for each condition.

Title: Supplementary Data 3.
Description: Paired two-sided t-tests to evaluate the significance of the differential performance of preservatives and extraction kits used with non-standardized healthy stool samples. N = 4 for each condition, considering both stool samples (Omni and Veg) cumulatively.

Title: Supplementary Data 4.
Description: Paired two-sided t-tests to evaluate the significance of the differential performance of extraction kits used with clinical samples stored in ZY preservative. N = 20 for BCoV M gene detection. N = 11 for N1 gene detection because samples that did not yield detectable target RNA across at least 1 extraction kit were excluded from analysis.

Title: Supplementary Data 5.
Description: Sequences of oligonucleotides used as primers and probes in this study.

Title: Supplementary Data 6.
Description: MIQE guidelines checklist.[46]

Title: Supplementary Data 7.
Description: dMIQE guidelines checklist.[47]
