# Supplementary material for: Standardized preservation, extraction and quantification techniques for detection of fecal SARS-CoV-2 RNA
Source: Nat Commun. 2021 Oct 1;12:5753. doi: 10.1038/s41467-021-25576-6 (PMC8486790; doi:10.1038/s41467-021-25576-6)
Supplement: Supplementary file 11 — Reporting Summary [file 41467_2021_25576_MOESM11_ESM.pdf]

nature portfolio

Corresponding author(s): Ami S. Bhatt

Last updated by author(s): 08/03/2021

Reporting Summary

Nature Portfolio wishes to improve the reproducibility of the work that we publish. This form provides structure for consistency and transparency in reporting. For further information on Nature Portfolio policies, see our Editorial Policies and the Editorial Policy Checklist.

Statistics

For all statistical analyses, confirm that the following items are present in the figure legend, table legend, main text, or Methods section.

n/a	Confirmed	
☐	☒	The exact sample size (*n*) for each experimental group/condition, given as a discrete number and unit of measurement
☐	☒	A statement on whether measurements were taken from distinct samples or whether the same sample was measured repeatedly
☐	☒	The statistical test(s) used AND whether they are one- or two-sided<br>*Only common tests should be described solely by name; describe more complex techniques in the Methods section.*
☐	☒	A description of all covariates tested
☐	☒	A description of any assumptions or corrections, such as tests of normality and adjustment for multiple comparisons
☐	☒	A full description of the statistical parameters including central tendency (e.g. means) or other basic estimates (e.g. regression coefficient) AND variation (e.g. standard deviation) or associated estimates of uncertainty (e.g. confidence intervals)
☐	☒	For null hypothesis testing, the test statistic (e.g. *F*, *t*, *r*) with confidence intervals, effect sizes, degrees of freedom and *P* value noted<br>*Give P values as exact values whenever suitable.*
☒	☐	For Bayesian analysis, information on the choice of priors and Markov chain Monte Carlo settings
☒	☐	For hierarchical and complex designs, identification of the appropriate level for tests and full reporting of outcomes
☐	☒	Estimates of effect sizes (e.g. Cohen's *d*, Pearson's *r*), indicating how they were calculated

Our web collection on statistics for biologists contains articles on many of the points above.

Software and code

Policy information about availability of computer code

Data collection	Design and Analysis Software Version 2.4.3, the native software for the QuantStudio PCR machine used for RT-qPCR, was used to determine Cq. QX Manager 1.2 Standard Edition was used to set thresholds and quantify ddPCR data.
Data analysis	We performed statistical analyses using R (version 4.0.0). Linear regressions were plotted using the "ggpubr" package (version 0.4.0). Paired T-tests were performed using the stats (version 4.0.0) and rstatix (version 0.7.0) packages

For manuscripts utilizing custom algorithms or software that are central to the research but not yet described in published literature, software must be made available to editors and reviewers. We strongly encourage code deposition in a community repository (e.g. GitHub). See the Nature Portfolio guidelines for submitting code & software for further information.

Data

Policy information about availability of data

All manuscripts must include a data availability statement. This statement should provide the following information, where applicable:

- Accession codes, unique identifiers, or web links for publicly available datasets
- A description of any restrictions on data availability
- For clinical datasets or third party data, please ensure that the statement adheres to our policy

The authors declare that all other data supporting the findings of this study are available within the paper and in the associated file, Supplementary Information 1.

Field-specific reporting

Please select the one below that is the best fit for your research. If you are not sure, read the appropriate sections before making your selection.

☒ Life sciences ☐ Behavioural & social sciences ☐ Ecological, evolutionary & environmental sciences

For a reference copy of the document with all sections, see nature.com/documents/nr-reporting-summary-flat.pdf

Life sciences study design

All studies must disclose on these points even when the disclosure is negative.

Sample size

Testing of RNA standards - Two stocks of the synthetic RNA standards from ATCC and NIST were used. Each of the stocks was processed by a different user to generate a five-point ten-fold dilution series. This dilution series was then assayed in quadruplicate RT-qPCR and duplicate ddPCR reactions. The concordance of results across these replicates provided confidence in replicability and eliminated the need for more samples/replicates.

Testing of preservatives and extraction kits using standardized stool: Two independent users tested all combinations of preservatives and extraction kits (9 combinations in total) using aliquots of stool spiked with two concentrations of ATCC RNA (10^3 and 10^4 copies/µL), two concentrations of BCoV attenuated vaccine (1:10 and undiluted), and one set of control samples with no RNA. These 45 combinations of samples were extracted by two different users in technical duplicates and assayed by duplicate RT-qPCR and singlet ddPCR reactions. Sample size was determined by the practicality of testing a large sample set across an exhaustive combination of methodological variables. We are reassured of the sample size given concordance of results. Finally, given the concordance of results from biological replicates from the same user, we limited the number of replicates to one per user in subsequent experiments.

Testing of preservatives and extraction kits using healthy stool: Two independent users tested all combinations of preservatives and extraction kits (9 combinations in total) using two different aliquots of stool spiked with ATCC RNA at 10^3 copies/µL, BCoV attenuated vaccine at 1:10 dilution, and one set of control samples with no RNA. These 27 combinations of samples were extracted by two different users and assayed by duplicate RT-qPCR and singlet ddPCR reactions. Sample size was informed by the consistency of results in the previous experiment with standardized stool, and practicality of testing a large sample set across an exhaustive combination of methodological variables. We are reassured of the sample size given concordance of results.

Testing of ZY and OG preservatives in real-world setting: Data from the analysis of 188 samples collected from COVID-19 outpatients as part of a clinical trial were used towards this study. Sample size was determined by the total number of prospective samples collected from patients.

Comparing the performance of extraction kits on ZY preserved clinical samples: We picked 20 random samples from a biobank of stool collected from COVID-19 positive outpatients on the day of enrollment in a clinical trial. We then extracted RNA from two aliquots of these samples both with and without an internal spiked-in BCoV control. This resulted in 40 stool samples extracted using 3 kits, and assayed by singlet ddPCR reactions. Sample size was determined by the practicality of testing a large sample set across an exhaustive combination of methodological variables. We are reassured of the sample size given concordance of results.

Data exclusions

No data was excluded in our work. Further, complete data are provided in Supplementary Information 1.

Replication

Testing of RNA standards - Two stocks of the synthetic RNA standards from ATCC and NIST were used. Each of the stocks was processed by a different user to generate a five-point ten-fold dilution series. This dilution series was then assayed in quadruplicate RT-qPCR and duplicate ddPCR reactions. In RT-qPCR reactions, all assays are concordant except - a) one out of a total of eight RT-qPCR reactions assaying the NIST RNA for the E gene at 10^4 RNA concentration failed to amplify likely due to an experimental error in the RT-qPCR assay, b) the detection of RdRP at the lowest concentration of 10^0 copies/µL. The poor efficiency of the primer/probe targeting the RdRP gene is consistent with previous literature and explained in the manuscript. In ddPCR reactions, all assays are concordant except the detection of the RdRP gene up to a concentration of 10^1 copies/µL and one our of four reactions targeting the E gene at 10^0 copies/µL.

Testing of preservatives and extraction kits using standardized stool: Two independent users tested all combinations of preservatives and extraction kits (9 combinations in total) using aliquots of stool spiked with two concentrations of ATCC RNA (10^3 and 10^4 copies/µL), two concentrations of BCoV attenuated vaccine (1:10 and undiluted), and one set of control samples with no RNA. These 45 combinations of samples were extracted by two different users in technical duplicates and assayed by duplicate RT-qPCR and singlet ddPCR reactions. RT-qPCR results are concordant to a whole number Cq value. ddPCR results are concordant within an order of magnitude of viral load.

Testing of preservatives and extraction kits using healthy stool: Two independent users tested all combinations of preservatives and extraction kits (9 combinations in total) using two different aliquots of stool spiked with ATCC RNA at 10^3 copies/µL, BCoV attenuated vaccine at 1:10 dilution, and one set of control samples with no RNA. These 27 combinations of samples were extracted by two different users and assayed by duplicate RT-qPCR and singlet ddPCR reactions. RT-qPCR results are concordant to a whole number Cq value and ddPCR results are concordant within an order of magnitude of viral load barring two exceptions. In the ddPCR assay, one out of four of the samples preserved in PBS and extracted using MM provided a low viral load, while the others failed. We anticipate that this is an experimental error yielding viral load just above the limit of detection of this assay. Further, we detected BCoV from the PBS sample extracted with the ZV kit in the RT-qPCR assay, albeit at a high Cq value, but not in the ddPCR assay. This is likely a false positive.

Testing of ZY and OG preservatives in real-world setting: Data from the analysis of 188 samples collected from COVID-19 outpatients as part of a clinical trial were used towards this study. This data was generated elsewhere and analysis did not work with replicates.

Comparing the performance of extraction kits on ZY preserved clinical samples: We picked 20 random samples from a biobank of stool

collected from COVID-19 positive outpatients on the day of enrollment in a clinical trial. We then extracted RNA from two aliquots of these samples both with and without an internal spiked-in BCoV control. This resulted in 40 stool samples extracted using 3 kits, and assayed by singlet ddPCR reactions. Therefore, this experiment did not include replicates.

Randomization

We did not randomize samples because we needed to test the same sample across multiple preservatives, extraction kits and PCR methods. Where we had replicates assay reactions, the replicate samples were assayed in different locations on the plate, to ensure there were no biases from sample location.

For comparing the performance of extraction kits on ZY preserved clinical samples, we picked 20 random samples from a biobank of stool collected from COVID-19 positive outpatients on the day of enrollment in a clinical trial.

Blinding

RT-qPCR and ddPCR allow the absolute measurement of viral load, in a manner that is completely devoid of subjectivity. Further, every assay plate included controls to measure the accuracy of the detection assay. Taken together, there was no need to blind the researchers since assays were thoroughly controlled to avoid biases.

Reporting for specific materials, systems and methods

We require information from authors about some types of materials, experimental systems and methods used in many studies. Here, indicate whether each material, system or method listed is relevant to your study. If you are not sure if a list item applies to your research, read the appropriate section before selecting a response.

Materials & experimental systems

n/a	Involved in the study
☒	☐ Antibodies
☒	☐ Eukaryotic cell lines
☒	☐ Palaeontology and archaeology
☒	☐ Animals and other organisms
☐	☒ Human research participants
☒	☐ Clinical data
☒	☐ Dual use research of concern

Methods

n/a	Involved in the study
☒	☐ ChIP-seq
☒	☐ Flow cytometry
☒	☐ MRI-based neuroimaging

Human research participants

Policy information about studies involving human research participants

Population characteristics

Samples from human subjects were used in three experiments:
1. Standardized stool sample from NIST was used. This includes samples from multiple healthy donors, homogenized into a single stool sample. Therefore, effects of population characteristics of donors are negated.
2. Non-standardized stool sample from healthy donors. This includes samples from one male healthy donor on a vegetarian diet and one female healthy donor on a gluten free omnivorous diet. The sample size here is not large enough to perform covariate analysis.
3. Samples and data from participants in the clinical trial. Detailed covariate analysis towards these are included in the parent study. The current study does not analyze relevant metadata.

Recruitment

Healthy volunteers were recruited for the stool donation study (through the IRB protocol listed below) using flyers in the University neighborhood. Participants for the clinical study were recruited through a different IRB protocol as described in Jagannathan et al, Nature Communications 2021. Briefly, subjects were recruited to this randomized controlled study of Peg-interferon-lambda for the treatment of mild COVID through flyers, online advertisement, advertisements at local care facilities, and other media outlets. Advertising was carried out in multiple languages that are commonly spoken in the region. We do not anticipate any self-selection bias.

Ethics oversight

Samples from healthy donors were collected as per Stanford IRB protocol #42043 (PI: Ami S Bhatt; Title : Genomic, Transcriptomic and Microbiological Characterization of Human Body Fluid Specimens).

Samples and data from patients were collected as per Stanford IRB protocol #55619 (PI: Upinder Singh, Prasanna Jagannathan; Title : A Phase 2 Randomized, Open Label Study of a Single Dose of Peginterferon lambda-1a (Lambda) Compared with Standard Supportive Care in Outpatients with Mild COVID-19).

Note that full information on the approval of the study protocol must also be provided in the manuscript.
